# Plastic Metallic Filling

**Published:** 1862-09

**Authors:** B. Wood


### PLASTIC METALLIC FILLING, AS A SUBSTITUTE FOR THE BASE METALS AND METALLIC OXIDES IN USE FOR FILLING TEETH.

BY DR. B. WOOD.

In 1860 a patent was granted me for fusible metal suitable for casting, and as a metallic cement and other purposes, requiring a metal melting at a very low temperature.

Of this metal the London Chemical News says:

"Lipowitz has made some experiments on the cadmium alloy described by Dr. Wood. (See Chemical News, vol. ii, p. 257.) It is permanently silver-white, and has a brilliant metallic luster; it is not so brittle or hard but that it may be obtained in thin leaves or flexible plates; it has a fine grained fracture, and may be filed without stopping up the file. In dry air it keeps its polish. It expands on cooling, but not so much as bismuth or antimony. Its specific gravity is from

9.4 to 9.41. It softens between 131° and 140° Fah., and near 140° becomes perfectly fluid. No change in the condition of the melted mass was observed on re-melting, after rapidly cooling the alloy. The above properties show that the alloy may be applied to some useful purposes. * * * * It may supersede all the quicksilver alloys for stopping teeth; it may be used as a solder wherever the metals soldered are not likely to be exposed to heat. * * * * The alloy is so easily fusible that it may be melted in a piece of paper over a spirit lamp." (Dinzler's Polytech. Jour., Bd. clviii. § 271 and 376, and Jour. Frank. Inst., vol. xli. p. 418.)

Having, in compliance with the demands of the case, prepared the metal in a *plastic* form, especially adapted to the purpose of tooth-filling, I desire to elicit the candid and impartial examination of the profession upon the subject.

When we see how extensively the base metals, amalgams, and metallic oxides are used for filling teeth, we can not be insensible to the demand for a material possessing the recommendations claimed for these; and it is surely of no small interest to the profession, if we can produce something in any way free from their defects, or possessing superior advantages. It is in vain that we repudiate the use of these baser materials. Dentists will still use them, and a large proportion of the community can not or will not bear the expense of *good* gold fillings; and it must be conceded that an imperfect filling with gold is less serviceable to the patient than a perfect filling with a baser metal. Since, therefore, it is impossible to control the profession and community as to the use of cheap materials, it becomes us to do what we can to improve upon them.

The stopping of teeth with substances rendered fluid or plastic by heat has, to a certain extent, long been in vogue. Formerly mastic, sulphur and various cements were used; and latterly gutta percha, Hill's stopping, etc., are resorted to. These, where not otherwise objectionable, are deficient in durability. As a more durable material, Newton's fusible metal, consisting of lead, bismuth and tin, and melting at the heat of boiling water, was at one time extensively employed. An objection to this, to start with, (apart from the general one of using the base metals in the mouth) was that it required too high a heat for its fusion. And although this particular objection was obviated by the addition of mercury to the alloy, yet this brought into requisition an obnoxious metal, and which, moreover, impaired the quality of the material, rendering it extremely fragile, besides the subsequent bad results attributed to it.

Nevertheless this compound, in the one form or the other, was in very general use until quite a late period, particularly in France. And it may, in passing, be of interest to quote the remarks upon it of some of the prominent dental authors who wrote at the time it was most in vogue. This fusible metal passed by the name of Darcet's metal, after its reputed discoverer, although the discovery is generally ascribed to Sir Isaac Newton, who flourished nearly a century earlier.

F. Maury (Treatise on the Dental Art, Paris, 1833,) speaks of certain cases of plugging where, he says, "we may advantageously use the fusible metal of Darcet, used for the first time by our confrère, M. Regnart, who has improved it by adding a tenth part of mercury. This composition consists of bismuth 8 parts; lead 5 parts; tin 3 parts. It is fusible at the temperature of boiling water. . . . . Many dentists use this article exclusively, the advantage of which we have made known to many of our confrères. We are much indebted to M. Regnart for this discovery."

M. Desirabode (Complete Elements of the Science and Art of the Dentist, Paris, 1845), having enumerated lead, tin, gold, platina, etc., as materials for plugging teeth, says: "But the metallic compound which is known as Darcet's metal is more used at the present day than any of the metals which we have named. . . . . This compound is rendered more fusible by the addition of a tenth of mercury; but this addition, to say the least, we regard as useless, first, because the preparation compounded according to M. Darcet's receipt is sufficiently fusible, and because the mercury has the effect of turning black sets of artificial teeth in the mouth, as also the teeth which are filled with it." After alluding to the "immense advantage" of its fusing at so low a temperature, and describing the mode of using it, he adds: "This metal, as will be readily perceived, accommodates itself better than any other to the different forms of the dental cavities; it is only to be regretted that its gray metallic color contrasts with the natural color of the teeth, that it can only be used for the lower teeth, and particularly when the cavities are upon the grinding surfaces of the larger molars; besides these objections, there are many persons who can not be made sensible of its manner of fusion, and who become alarmed at the necessary preparation of heating the instrument, expecting it to produce pain, which we will say, *en possant*, is frequently quite acute." (This last remark, it will be perceived, conflicts with what was before said about the metal being "sufficiently fusible" without the addition of mercury.)

Lefoulon, writing about the same date, says, (Theory and Practice of Dental Surgery, Paris, 1841.) "This fusible metal offers several great advantages. It forms a compact, homogeneous mass without fissures, which will not admit the saliva or buccal humors to penetrate it. Nevertheless, it requires the use of a degree of heat which, though not sufficient to burn the teeth and surrounding parts, may yet be sufficient to inflame the latter and dry the enamel until it cracks. [He is speaking of the formula as originally prepared, without the addition of mercury.] Besides, it requires the utmost skill, acquired by long experience, to seize the precise point of temperature, not to suffer it to fall short of the degree desired, and thus defeat the operation; and on the other side, not to suffer it to be exceeded, so as to burn the organ we wish to save, or the surrounding parts." . . . . [Having objected to amalgams, in reply to the plea for it that it contains less mercury than what is added to the fusible metal, he says]:

"When we have had occasion to use the fusible metal, we have taken good care not to add the mercury, for the reason that [as is the case] after the employment of the amalgam of silver and mercury, we have observed sorrowful results from the use of it to the health of the gums and the alveolar periosteum."

We may add here what Dr. Harris (Dictionary of Dental Surgery) says on the subject, as embodying the objections of recent authorities: "DARCET'S METAL.—An alloy fusible at 212° F., composed of 8 parts bis., 5 lead, 3 tin. It was at one time much used for filling teeth, especially of the lower jaw, into the cavities of which, while in a fused state, it can be easily introduced. The use of it, however, for this purpose was soon abandoned, for the reason that the temperature at which it had to be applied could not in all cases be borne, and it frequently caused inflammation of the lining membrane. Besides, it was found that it shrank from the walls of the cavity in cooling, so as to admit the secretions of the mouth, consequently, it did not prevent the recurrence of disease."

We have here the drift of what has been urged *pro* and *con.* in respect to this metal. But the great practical difficulty in the way of its successful employment grew out of the tendency of the liquified mass to assume the spherical form, refusing to remain until congealed in contact with portions of the walls of the cavity, so that it was hardly practicable to secure a filling but that, at certain points, interstices would be left between the metal and dentine for the absorption of fluids. It was really this that, upon after examination, gave rise to the opinion that the metal shrank from the walls of the cavity in cooling, whereas the fact was, that in consequence of the centripetal attraction of its particles, it never came in close contact at those points from which it was supposed to have shrunken in congealing, for the mass rather expands in congealing. But the difficulty was none the less fatal to perfect and successful fillings. This, in connection with the difficulty of introducing fillings with it at all, except in favorable cases of crown cavities in the lower jaw, caused it to give way to the more easily manipulated amalgams, which soon came in vogue.

By means, however, of the cadmium alloy above described, we have a material which, while well suited for a durable filling, and melting at a temperature sufficiently low, without any addition of mercury, completely obviates the objections just referred to. In the plastic form in which I have succeeded in producing it, it is as easy of application to the teeth of the superior maxilla and to lateral cavities, as to common cavities of the lower teeth. For working, it requires a temperature no greater than that required for applying mastic or "Hill's stopping," or indeed than the heat at which gold is introduced by some dentists who "anneal" their foil; while at this working temperature it remains plastic, so that it may be *packed* securely to every part of the cavity. It can be managed with greater facility than even amalgams or the cement of oxichloride of zinc, and by skillful manipulation may be introduced into a cavity in the length of time required to prepare these for introduction, while it is free from the great objections urged against them. To amalgam, a mixture of mercury with filings of an alloy of tin and silver, or sometimes other metals, it is objected that the mercury becomes partly disengaged from the compound, and even if not converted into oxide detrimental to health, permeates the dentinal tubes, and so blackens the tooth. Of the zinc preparation, under its various forms and names, consisting essentially of powdered oxide of zinc made into a paste with liquid chloride of zinc (a solution of metallic zinc in muriatic acid) it may be urged that it injures the tooth directly, in consequence of the affinity of the acid for its phosphate of lime; besides that, after becoming thoroughly hardened in the tooth, the material is still comparatively soft and friable, so as to be worn away by the attrition of mastication.

The plastic metallic filling forms a durable plug. It is harder than block tin, and will probably outwear any material now generally used in the teeth, except *solid* gold fillings. It has the advantage of the susceptibility of being built out in any form from the cavity of a tooth. And it is, to say the least, not more liable to oxidize or tarnish than tin under like conditions.

The objections, if any except that of using the base metals for this purpose, and which is alike applicable in respect to tin, must hold against its constituents. These, as is well known, consist of cadmium, tin, bismuth and lead. To the first three I presume little can be objected. Cadmium has been recently used to some extent for filling teeth, combined with mercury, forming the amalgam recommended by Dr. Evans, of Paris, although its supposed advantages as a substitute for silver in such connection has not been demonstrated; and the profession in this country have at present generally settled upon an alloy of silver and tin as the basis for these compounds. Bismuth may be regarded as innocuous as tin. Lead, from time immemorial down to quite a late period, has been the principal substance used for filling teeth. The French word employed to designate this operation is *plombage* (*leading*) from the chief substance resorted to for the purpose until within a score of years. Although its salts when taken internally, in comparatively small quantity, are injurious to health, yet we do not find this result attributed to it by those who raised objection to it at the time it was in vogue.

Maury, who wrote about 1830, having mentioned lead, tin, gold, platina, etc., says: "Lead, of all the substances which we have enumerated, is most generally used for plugging teeth," but adds that, "as this metal oxidizes and becomes black," he prefers tin. His contemporaries of a little later date urge the same objections, but without giving intimation of injurious effects upon the system having resulted from its use; although subsequent writers speak of the possibility of such results. If in such quantity as was liable to be oxidized or abraded from fillings, it was capable of affecting the constitution, it ought to have been noticed at the time when the metal was in the mouths of every one resorting to the operation, especially when the writers were not unobservant of any bad effects resulting from the use of amalgams and other substances. If lead itself, although easily worn off and oxidized in the mouth, was not observed to produce an appreciable effect upon the system when it was universally in use for filling teeth, there really would not seem to be much ground for objection to it on this score, as combined with and shielded by innocuous constituents in a compound not easily oxidized or worn away.

If desired, the surface of these fillings could be protected by welding over it a thin plate of tin or of gold, which may be readily done.

It may be remarked that the addition of mercury to this compound would be less objectionable than its use in connection with either silver, etc., or with the old fusible metal, because it enters into chemical combination with the cadmium, and the strong affinity of this metal for it holds it as a permanent constituent in the compound. Hence it is not liable to disengage itself, whether by exudation or evaporation. Whereas, in the other mixtures, the mercury does not enter into such intimate combination—but is rather held mechanically—whence its tendency to disengage itself. [Instance of mercury rubbing off from the surface of alloys, and evaporating when mixed into a paste with filing, and leaving them in process of time, as at first.]

---

The ideas set forth in this article are certainly worthy of attention by the dental profession, and especially by those who find it necessary to use a more economical material for filling teeth than gold. We think this alloy quite as good in any respect as tin, and in some, perhaps, much better. In the facility with which it can be introduced and manipulated, it is certainly much superior to tin foil; and then it is in a solid mass when introduced. We intend to try it. We presume Dr. Wood would furnish it to any who may apply for it. ED. REG.

